# Probiotics, Prebiotics, Synbiotics, and Paraprobiotics as a Therapeutic Alternative for Intestinal Mucositis

**DOI:** 10.3389/fmicb.2020.544490

**Published:** 2020-09-17

**Authors:** Viviane Lima Batista, Tales Fernando da Silva, Luís Cláudio Lima de Jesus, Nina Dias Coelho-Rocha, Fernanda Alvarenga Lima Barroso, Laisa Macedo Tavares, Vasco Azevedo, Pamela Mancha-Agresti, Mariana Martins Drumond

**Affiliations:** ^1^Laboratório de Genética Celular e Molecular (LGCM), Departamento de Biologia Geral, Instituto de Ciências Biológicas, Universidade Federal de Minas Gerais (UFMG), Belo Horizonte, Brazil; ^2^Faculdade de Minas, FAMINAS-BH, Belo Horizonte, Brazil; ^3^Centro Federal de Educação Tecnológica de Minas Gerais (CEFET/MG), Departamento de Ciências Biológicas, Belo Horizonte, Brazil

**Keywords:** lactic acid bacteria, chemotherapy, intestinal inflammation, treatment, mucosite

## Abstract

Intestinal mucositis, a cytotoxic side effect of the antineoplastic drug 5-fluorouracil (5-FU), is characterized by ulceration, inflammation, diarrhea, and intense abdominal pain, making it an important issue for clinical medicine. Given the seriousness of the problem, therapeutic alternatives have been sought as a means to ameliorate, prevent, and treat this condition. Among the alternatives available to address this side effect of treatment with 5-FU, the most promising has been the use of probiotics, prebiotics, synbiotics, and paraprobiotics. This review addresses the administration of these “biotics” as a therapeutic alternative for intestinal mucositis caused by 5-FU. It describes the effects and benefits related to their use as well as their potential for patient care.

## Introduction

Cancer is a disease characterized by uncontrolled proliferation of cells with cellular differentiation properties, having the capacity to invade tissues and organs and spread to other regions of the body, causing metastases (World Health Organization [WHO], 2018). This disease is the second leading cause of death globally, according to the World Health Organization, accounting for an estimated 9.6 million deaths in 2018; lung (1.76 million deaths), colorectal (862,000 deaths), stomach (783,000 deaths), liver (782,000 deaths), and breast cancer (627,000 deaths) are the most common types and have the highest mortality rates (World Health Organization [WHO], 2018).

Despite the high incidence and mortality rates, when identified early, cancer is a potentially curable and treatable disease. Treatment may be done through surgery, chemotherapy, radiotherapy, or bone marrow transplantation, depending on the type of cancer, degree of tumor aggressiveness, as well as the patient’s physical and immunological status. It is often necessary to combine more than one type of treatment to achieve satisfactory results (World Health Organization [WHO], 2018).

Antineoplastic chemotherapy consists of the use of drugs that destroy cancer cells, inhibit their growth, and prevent their spread by targeting DNA or critical processes involved in cell division (Guichard et al., 2017; Shields, 2017). The traditional chemotherapeutics are classified according to their mechanisms of action, including antimetabolites, microtubule-targeting agents, topoisomerases, and antibiotics (Shields, 2017). The therapeutic arsenal mostly used in the treatment of neoplasms include oxaliplatin, irinotecan, capecitabine, cisplatin, methotrexate, 5-fluorouracil (5-FU), and FOLFIRI (an association of 5-fluorouracil, irinotecan, and leucovorin), among others (Nussbaumer et al., 2011; Cassidy and Syed, 2017; Guichard et al., 2017).

**FIGURE 1:**
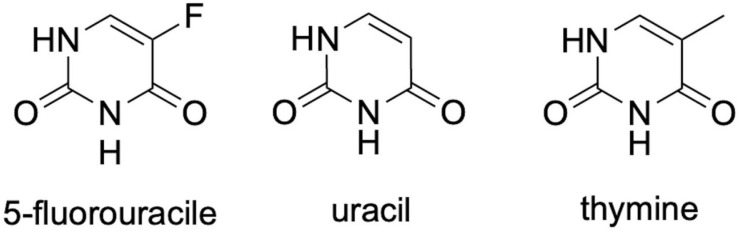
Chemical structure of 5-fluorouracil (5-FU) and its analogs uracil and thymine. All three structures differ in the radical present at the structure’s fifth position.

The medication 5-FU is highlighted among the chemotherapeutic alternatives and has been mainly used in the treatment of advanced types of cancer, such as colorectal cancer, as well as malignant head and neck cancer, breast, stomach, and some skin cancers (Longley et al., 2003; Martins and Wagner, 2013; Cassidy and Syed, 2017; Guichard et al., 2017). This drug is an analog of uracil and thymine (Figure 1), which is metabolized in the liver, producing many metabolites. One of them binds to and inhibits the enzyme thymidylate synthase and, consequently, ends up interfering with DNA synthesis and cell division (see the *Mechanism of Action of 5-FU* section). On the other hand, this drug can act by the incorporation of its metabolites into the DNA and/or RNA of these cells (Sonis, 2004), which impedes their normal functioning and induces apoptosis (Longley et al., 2003; Miura et al., 2010).

However, 5-FU’s non-specific mechanism of action results in side effects such as nausea, cardiotoxicity, leukopenia, alopecia, myelosuppression, diarrhea, and oral and intestinal mucositis (Duncan and Grant, 2003; Soveri et al., 2014; Thomas et al., 2016; Cinausero et al., 2017). Intestinal mucositis is the most prevalent side effect of 5-FU therapy (50–80% of reported cases) and one of the main limiting factors for continuing treatment (Kim et al., 2015).

Mucositis is an inflammation of the gastrointestinal tract (GIT), with symptoms that include diarrhea, abdominal pain, bleeding, fatigue, malnutrition, electrolyte imbalance, and infections, causing complications that may be life threatening (Sonis, 2004; Touchefeu et al., 2014; Kim S. et al., 2018). The cytotoxic effects of 5-FU in the GIT cells are a severe problem for oncological therapeutics, as they decrease the patient’s ability to tolerate treatment, affecting the quality of life, directly influencing the success of therapy (Jamali et al., 2018).

Within this context, therapeutic alternatives have been sought as a means to prevent or ameliorate intestinal mucositis. Among these alternatives, the most promising are the use of probiotics [“*live microorganisms which when administered in adequate amounts confer a health benefit on the host”* (FAO/WHO, 2001)], prebiotics [*“a substrate that is selectively utilized by host microorganisms conferring a health benefit”* (Gibson et al., 2017)], synbiotics [*“a mixture of probiotics and implantation of live microbial dietary supplements in the GIT, by selectively stimulating the growth and/or activating the metabolism of one or a limited number of health-promoting bacteria, and thus improving host welfare*” (Gibson and Roberfroid, 1995)], paraprobiotics, and postbiotics, which can be defined as non-viable microorganisms, cell fractions or cell metabolites, bacteriocins, organic acids, and enzymes (Rad et al., 2020).

In this review, we address the evidence for the suitability of probiotics, prebiotics, synbiotics, and paraprobiotics as a therapeutic alternative for intestinal mucositis caused by the antineoplastic drug 5-FU.

## Mechanism of Action of 5-FU

The drug 5-FU is an antimetabolite analogous to uracil, which differs by the substitution of a hydrogen atom with fluorine at the fifth position of the uracil molecule. Developed in the 1950s and introduced in cancer therapy to inhibit cell division and proliferation of cancer cells, this substance is among the class of antineoplastic drugs with a vast spectrum of action in oncological practice, being widely used for the treatment of a variety of tumors (Thomas et al., 2016; Kato et al., 2017).

**FIGURE 2:**
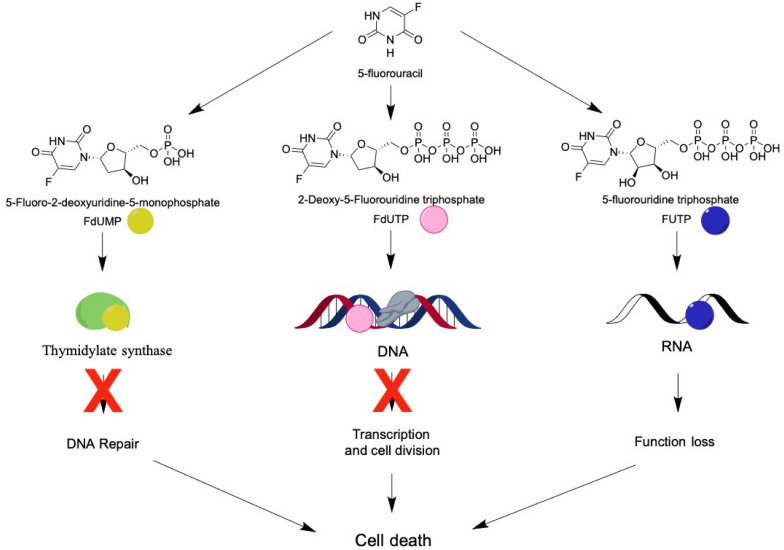
5-FU’s metabolites and their molecular targets. 5-FU is intracellularly metabolized into fluorodeoxyuridine monophosphate (FdUMP), which binds to the enzyme thymidylate synthetase, resulting in decreased production of dTTP and dATP and blocking cell repair; or into fluorodeoxyuridine triphosphate (FdUTP) and binds to the DNA, inhibiting duplication and transcription; or into 5-fluorouridine triphosphate (FUTP) and binds to RNA, leading to a loss of function. All three metabolites cause damage to the cell on a genomic level, culminating in cell death.

To control the abnormal proliferation of cancer cells, 5-FU enters into the cells through facilitated transport, which is the same mechanism involved in its intracellular conversion into active metabolites [fluorodeoxyuridine monophosphate (FdUMP), fluorodeoxyuridine triphosphate (FdUTP), and 5-fluorouridine triphosphate (FUTP)]. These metabolites may exhibit three different mechanisms of action: (1) FdUMP inhibits the activity of the enzyme thymidylate synthase causing an imbalance in the pool of nucleotides, consequently decreasing the concentration of the deoxynucleotides dTTP and dATP, essential for DNA repair; (2) FdUTP binds to the DNA structure, inhibiting its synthesis, blocking cell division; and (3) FUTP can be incorporated into RNA, damaging it, leading to functional loss and cell death (Longley et al., 2003; Zhang et al., 2008; Miura et al., 2010; Figure 2).

Clinical evidence of patients undergoing oncologic therapy with 5-FU shows that the effects of this chemotherapy vary among users. From 20 to 40% of the patients treated with the standard dose of this drug (10–15 mg/kg body weight, for 3–4 days intravenously) develop some degree of mucositis, and about 80–100% of the patients treated with high doses (350–500 mg/kg body weight) develop GIT problems (Crombie and Longo, 2016; Cinausero et al., 2017).

## Effects of 5-FU on the Gastrointestinal Tract

In addition to having a digestive and nutrient absorption role, the GIT mucosa acts as a physical and immunological barrier, having the ability to defend the body against potentially harmful agents that can trigger inflammatory responses in the intestine (Salvo Romero et al., 2015; König et al., 2016). The intestinal barrier is categorized according to the various levels of protection, as well as the location and nature of its cellular and extracellular components (Vancamelbeke and Vermeire, 2017). These include mainly the mucus layer associated with the commensal microbiota of the gut, antimicrobial peptide and immunoglobulin A (IgA) secretion, the monolayer of specialized epithelial cells (enterocytes, Paneth cells, goblet cells, stem cells, and enteroendocrine cells), and the lamina propria, a specialized connective tissue in which innate and adaptive immune cells reside, such as T cells, B cells, dendritic cells (DCs), macrophages, neutrophils, eosinophils, and the newly discovered innate lymphoid cells (ILCs) (Vancamelbeke and Vermeire, 2017).

Although the intestinal barrier plays an essential role in the body’s homeostasis, it is susceptible to 5-FU oncologic therapy (Yu, 2013). The intestinal mucositis caused by 5-FU mainly affects the small intestine (duodenum, jejunum, and ileum), characterized by inflammation, loss of intestinal structure and functionality, villous atrophy, goblet and Paneth cell degeneration, reduction in mucin secretion, increased intestinal permeability, cell death, polymorphonuclear cell infiltration, and increased production of proinflammatory cytokines, such as interleukin-1β (IL-1β), IL-6, and tumor necrosis factor-α (TNF-α), mucosal tissue exposed to infection, and alteration of the intestinal microbiota composition (Chang et al., 2012; Lee, 2014).

**FIGURE 3:**
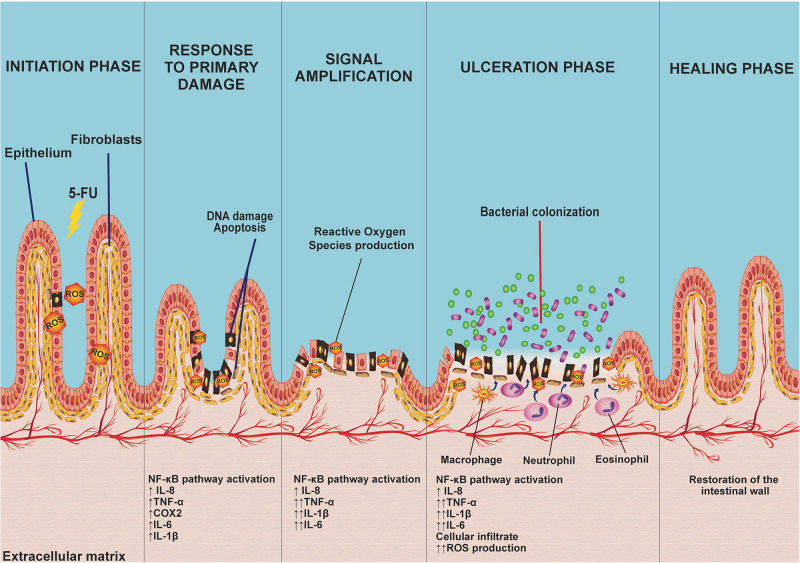
The five phases of 5-FU-induced intestinal mucositis: The initiation phase occurs when the intestinal mucosa is first exposed to the toxicity of 5-FU, promoting DNA damage and inducing the production of reactive oxygen species (ROS). Consequently, this activates several signaling transduction pathways (response to primary damage) such as the NF-êB pathway, related to the induction of several inflammatory mediators [interleukin-8 (IL-8), tumor necrosis factor-α (TNF-α), cyclooxygenase-2 enzyme (COX-2), IL-6, and IL-1β)] that play an important role in mucosal toxicity, causing signal amplification via a positive feedback mechanism, activating pathways that increase cytokine production as well as oxidative stress, exacerbating the lesion, progressively destroying the mucosa leading to an ulceration phase. Finally, spontaneous ulcer healing, characterized by cell proliferation and differentiation on average 3–4 days after the end of chemotherapy treatment, leads to mucosal restoration.

The pathology of mucositis can be divided into five phases (initiation, response to primary damage, signal amplification, ulceration, and healing) (Sonis, 2004; Figure 3). The *initiation phase* occurs when the intestinal mucosa is exposed to 5-FU, which promotes DNA/RNA damage, either because it binds directly to these biomolecules or through the oxidative stress caused by reactive oxygen species (ROS) production. These factors induce tissue damage (Sonis, 2004; Villa and Sonis, 2015; Cereda et al., 2018), which activates several signal transduction pathways, such as nuclear factor κB (NF-κβ) pathway signaling. This situation leads to the induction of various inflammatory mediators, such as IL-8, TNF-α, cyclooxygenase-2 enzyme (COX-2), IL-6, and IL-1β, among others, that are responsible for mucosal toxicity (Sonis, 2004; Cinausero et al., 2017).

The recruitment of these proinflammatory cytokines acts indirectly on *signal amplification* (amplification phase) via a positive feedback mechanism, activating pathways that increase proinflammatory cytokine production (TNF-α, IL-1β, and IL-6), as well as oxidative stress. The increase in the production of these factors initiates a cascade of reactions that leads to the activation of matrix metalloproteinases, resulting in tissue damage or an increase in TNF-α production, exacerbating the initial lesion (Sonis, 2004).

The progressive destruction of the mucosa culminates in an *ulceration phase*, which occurs when loss of integrity and function of the epithelium occurs. At this stage, there are symptomatic lesions that, apart from being prone to pathogenic bacterial colonization, stimulate the activation and infiltration of defense cells, including macrophages, neutrophils, and eosinophils, in the intestinal mucosa. These cells increase the production of oxidant compounds, resulting in an increase in the depth of intestinal ulcers, consequently increasing bacterial translocation (Villa and Sonis, 2015; Cinausero et al., 2017; Cereda et al., 2018).

Finally, *the healing phase* is characterized by cell proliferation and differentiation. This phase occurs, on average, 3–4 days after the last chemotherapy treatment, leading to restoration of the mucosa (Sonis, 2004; Villa and Sonis, 2015).

## Effects of 5-FU on Intestinal Microbiota

In addition to causing structural damage to the intestinal epithelium, the mucositis caused by chemotherapeutic agents has a crucial influence on the intestinal microbiota (van Vliet et al., 2010). The GIT has a complex ecological population, constituted by more than a thousand different species of microorganisms, though their distribution varies along the GIT (Mowat and Agace, 2014; Rajilić-Stojanović and de Vos, 2014); low concentrations and bacterial diversity (up to 10^3^ CFU/ml) are found in the upper GIT (stomach, duodenum, jejunum, and proximal ileum) (Walter and Ley, 2011). A larger number of bacteria (10^9^–10^12^ CFU/ml) reside in the lower compartments of the GIT (distal ileum and colon), which constitutes, to date, the habitat with the highest known microbial density (Mowat and Agace, 2014; Jandhyala et al., 2015; Thursby and Juge, 2017). Due to the low oxygen tension in the colon, the most prevalent bacterial groups consist of anaerobic species, such a *Clostridia*, *Enterobacteria*, *Enterococcus*, *Bacteroides*, *Bifidobacteria*, *Fusobacteria*, *Lactobacilli*, *Peptococci*, *Peptostreptococci*, *Prevotellaceae*, *Roseburia*, *Ruminococci*, and *Verrucomicrobia* (Simon and Gorbach, 1982; Bäckhed et al., 2005; Mowat and Agace, 2014).

The intestinal microbiota acts through several mechanisms to maintain the homeostasis of the organism, living in mutuality with the host, benefiting from the nutrient-rich environment offered by the organism and, in exchange, performing innumerable beneficial functions, including elimination of pathogens, production of vitamins and short-chain fatty acids (SCFA), as well as modulation of the enteric and systemic immune systems (Lane et al., 2017; Thursby and Juge, 2017). However, when this mutualism becomes unbalanced, the intestinal microbiota can contribute to the onset of infectious diseases, chronic inflammation, and autoimmune diseases (de Oliveira et al., 2017).

The commensal microbiota, such as *Bifidobacterium infantis* and *Bacteroides thetaiotaomicron*, have been shown to decrease NF-κB activation, decreasing levels of endotoxins and of plasma proinflammatory cytokines (Stringer et al., 2009). Studies have demonstrated that treatment with 5-FU alters the relative abundance of several genera of the intestinal microbiota, such as *Clostridium*, *Lactobacillus*, *Enterococcus*, *Bacteroides*, *Staphylococcus*, *Streptococcus*, and *Escherichia* (Stringer et al., 2009). Thus, disrupted homeostasis of the intestinal microbiota can affect the mucosal immune system due to an imbalance between the production of pro- and anti-inflammatory mediators, resulting in intestinal inflammation (Autenrieth and Baumgart, 2017; Holleran et al., 2017).

Given the possibility that intestinal mucositis is closely related to intestinal microbiota dysbiosis (von Bültzingslöwen et al., 2003; Yu, 2018), probiotic microorganisms have been presented as an alternative treatment due to their beneficial properties in the GIT. Given these characteristics, several studies have shown that probiotics can be an effective therapeutic alternative for the reduction of antineoplastic-induced intestinal mucositis.

## Probiotics

In order, to be considered a probiotic and be able to exert health benefits for the host, microorganisms must have some specific attributes, such as being capable of remaining viable during transport and storage, and tolerating the low pH of the gastric lumen and the action of bile, and pancreatic and intestinal secretions. Many probiotics are able to colonize the GIT and stimulate the immune system (Wang M. et al., 2016; Mokoena, 2017). Furthermore, resistance to antibiotics in probiotic strains should be analyzed in order to assess their safety, as well as the level and the source of this resistance (Zhang et al., 2018). Intrinsic resistance is unlikely to spread horizontally between bacteria (Mathur and Singh, 2005), while acquired resistance could be transferred to other organisms, including pathogens, representing a potential risk to the health of the host (van Reenen and Dicks, 2011). The most well-studied and characterized probiotics belong to the lactic acid bacteria (LAB) group. However, other microorganisms also present probiotic properties, such as some *Saccharomyces* spp., and bacteria of the genera *Bifidobacterium* and *Faecalibacterium* (Pot et al., 2013; Bastos et al., 2016; Chang et al., 2019).

LAB mainly include the genera *Lactobacillus*, *Leuconostoc*, *Lactococcus*, *Pediococcus*, and *Streptococcus*, among others, and constitute a group of Gram-positive microorganisms, anaerobic or aerotolerant, non-spore forming, resistant to low pH, and able to produce lactic acid as the final product of the fermentation of carbohydrates (Wang Y. et al., 2016; Mokoena, 2017; Plavec and Berlec, 2019). Furthermore, these bacteria have been used for a long time in several industrial processes for the production of fermented foods, such as cheese, yogurts, etc. (Soccol et al., 2010), and they frequently present probiotic properties. Additionally, these organisms have been explored for protein heterology production and as live delivery systems for gene and biotherapeutic vaccines, with potential applications for the treatment and prevention of various pathological conditions, in both human and veterinary medicine (Carvalho et al., 2017; Gomes-Santos et al., 2017; LeCureux and Dean, 2018; Kuczkowska et al., 2019).

## Mechanisms of Action of Probiotics

**FIGURE 4:**
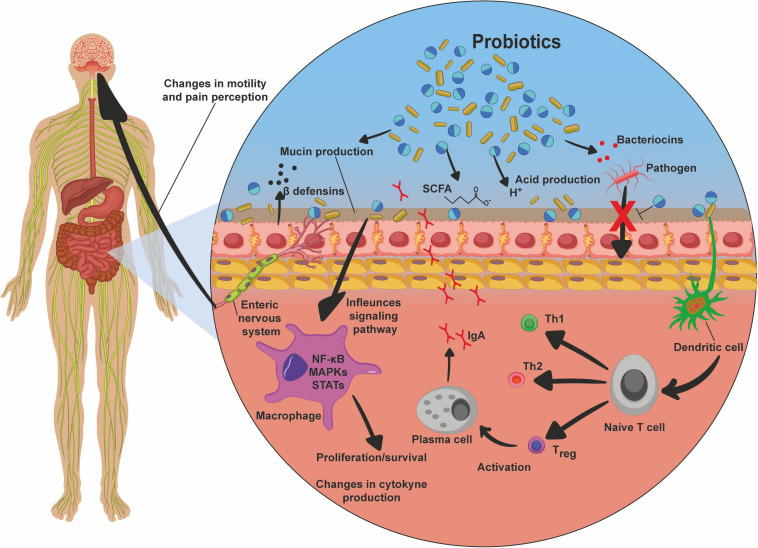
Probiotic mechanisms of action. Probiotics, after they reach the intestines, promote beneficial effects to the host by various mechanisms. These include mucin production, production of bacteriocins, acids, and short-chain fatty acids (SCFA), which are responsible for the inhibition of pathogens, inhibition of bacterial translocation, and inhibition of pathogens due to competition for receptors and nutrients. There is also stimulation of dendritic cells, which in turn induce the differentiation of T cells into Th1, Th2, and Treg, the latter being responsible for maturation of plasma cells, and thus immunoglobulin A (IgA) production and secretion; stimulation of beta-defensin production; inhibition of signaling pathways, such as nuclear factor κB (NF-kB), MAPK, and STAT, promoting proliferation and survival of the cells; changes in cytokine production profile, enhancing the production of anti-inflammatory cytokines and inhibiting proinflammatory factors; and interaction via the enteric nervous system with the central nervous system, promoting changes in intestinal mobility and pain perception.

Studies have shown that benefits for human health are attributed to consumption of probiotics, mainly for GIT diseases (Fedorak et al., 2015; Acurcio et al., 2017), though also for other diseases, including osteoporosis (Collins et al., 2018), cancer (Zaharuddin et al., 2019), obesity and type 2 diabetes (Saez-Lara et al., 2015; Wang et al., 2017; Hsieh et al., 2018), depression (Wallace et al., 2020), and atopic dermatitis (Rather et al., 2016). In this context, the main mechanisms of action described for these microorganisms in the host include: (i) colonization and regulation of a dysbiotic intestinal microbiota (Shi et al., 2017); (ii) protection of the epithelial barrier by maintaining tight junction integrity (Blackwood et al., 2017); (iii) induction of mucin production (Aliakbarpour et al., 2012) and B-cell-secreting IgA, which are important defense mechanisms necessary to maintain epithelial integrity and to protect the intestine from the external environment; (iv) increasing adherence to the intestinal mucosa and inhibiting of concomitant pathogen adherence based on competition for available nutrients and sites of mucosal adhesion (Collado et al., 2010; Monteagudo-Mera et al., 2019); (v) competitive exclusion of pathogenic microorganisms, such as *Staphylococcus aureus* and *Salmonella typhimurium* (Halder et al., 2017; Plaza-Díaz et al., 2017); (vi) production of antimicrobial substances such as acetic and lactic acids, and bacteriocins, which have strong inhibitory effects against Gram-negative bacteria and have been considered as the main antimicrobial compounds produced by probiotics against pathogens (Alakomi et al., 2000; De Keersmaecker et al., 2006; Makras et al., 2006; Bermudez-Brito et al., 2012; Mokoena, 2017; Gaspar et al., 2018; Castilho et al., 2019); (vii) production and secretion of metabolites of SCFAs with anti-inflammatory properties, such as acetate, propionate, and butyrate, which exert beneficial effects on intestinal and immune cells, being important compounds for cell proliferation, cell differentiation, and gene expression, and they are signaling molecules of immunological pathways; butyrate is the primary energy source of colonocytes, and it has an epithelial barrier function; SCFAs can also induce expression of the anti-inflammatory cytokine IL-10, inhibiting inflammatory responses (Parada Venegas et al., 2019); (viii) inhibition of the activation of the NF-κB signaling pathway (Kaci et al., 2011; Gao et al., 2015); (ix) interaction with the gut–brain axis via the production of metabolites such as γ-aminobutyric acid (GABA) (Kim N. et al., 2018); and (x) modulation of the host’s innate and/or adaptive immune system responses through interaction with epithelial cells, dendritic cells, monocytes, macrophages, and lymphocytes (Azad et al., 2018). In addition, probiotics can act by inducing host autophagy to attenuate oxidative stress-induced intestine injury (Wu et al., 2019; Figure 4).

Thus, due to the numerous possible pathways in which probiotics could be involved, their study as therapeutics of various diseases, especially those related to the GIT, is of particular importance.

## Effects of Probiotics on Intestinal Mucositis

The proposed mechanisms of action for the beneficial effects of probiotic microorganisms in diseases affecting the GIT are diverse, heterogeneous, strain specific, and depend on the quantity of probiotics used (Plaza-Díaz et al., 2017). Since the immunomodulatory and anti-inflammatory effects reported for LAB, as well as other probiotics, are strain dependent, it is necessary to identify and characterize species and strains with probiotic potential and investigate their effects on different targets or diseases (Plaza-Díaz et al., 2017). Table 1 presents the main findings for the effects of probiotics on intestinal mucositis.

**TABLE 1:** Effects of probiotics, prebiotics, synbiotics, and paraprobiotics in intestinal mucositis.

	**Effects of intestinal mucositis**	**References**
**Probiotics strain**		
*Lactobacillus acidophilus*	Inhibited nuclear factor κB (NF-κβ) (NF-κB) pathway signaling Regulated levels of the proinflammatory cytokines [tumor necrosis factor-α (TNF-α), interleukin-1β (IL-1β), and the C-X-C motif chemokine ligand (CXCL)] Reversed gastrointestinal dysmotility, increased gastric emptying and intestinal transit	Justino et al., 2015
*Lactobacillus acidophilus A4*	Stimulated the overexpression of mucin genes (MUC2 and MUC5AC) Reduced myeloperoxidase (MPO) activity Reduced expression of proinflammatory cytokine IL-1β	Oh et al., 2017
*Lactobacillus casei* variety *rhamnosus* (Lcr35)	Reduced production of proinflammatory cytokines (TNF-α, IL-1β, IFN-γ, IL-10, and IL-6). Attenuated the loss of goblet cells, decreased Firmicutes and increased Bacteroidetes Reduced the frequency of diarrhea Restored villus/crypt ratio	Yeung et al., 2015; Chang et al., 2018
*Bifidobacterium bifidum* G9-1	Attenuated histopathological alteration, with decrease cell infiltrate in crypts Regulated intestinal microbiota (decrease Firmicutes and increase Bacteroidetes abundance) Reduced the concentrations of proinflammatory cytokines TNF-α and IL-1β and MPO activity Reduced diarrhea and interrupt weight loss	Kato et al., 2017
*Bifidobacterium infantis*	Improved the histologic parameters, ameliorating mucosal damage Reduced Th1 and Th17 cells, and increased CD4 + CD25 + Foxp3 + Tregs response	Mi et al., 2017
Association*: (B. breve, L. acidophilus, L. casei, and Streptococcus thermophilus)*	Reduced neutrophil infiltration, proinflammatory cytokines (TNF-α, IL-4, and IL-6), and intestinal permeability Restored of the intestinal epithelium architecture	Tang et al., 2017
Association*: (L. acidophilus, L. paracasei, L. rhamnosus, and B. lactis)*	Prevented epithelial injury in intestinal mucositis, with an increase in the villus/crypt ratio Reduced the malondialdehyde (MDA), MPO, TNF-α, and IL-6 levels Increased glutathione (GHS) levels in the duodenum and jejunum sections	Quaresma et al., 2019
Association*:* Whey protein isolate, to skim milk fermented by *L. casei* and *Propionibacterium freudenteichii*	Ameliorated histological scores and prevented villus shortening Reduced weight loss and degeneration of goblet cells	Cordeiro et al., 2018
*Lactobacillus delbrueckii* subsp. *lactis* CIDCA 133	Prevented body mass loss Inhibited length reduction of the intestine caused by 5-fluorouracil (5-FU) Restored histopathological damage Reduced inflammatory parameters: neutrophil, eosinophil, leukocyte infiltrate reduction, and immunoglobulin A (IgA) secretion Reduced intestinal permeability	De Jesus et al., 2019
*Saccharomyces boulardii*	Reduced cells apoptosis and inflammatory factors (nitrite concentration, neutrophil infiltrate TNF-α, IL-1β cytokines, and CXCL-1 chemokine) Improved the intestinal functions such as gastric emptying, gastrointestinal transit, absorption, and intestinal permeability Modulated the expression of TLR2, TLR4, MyD88, NF-κB extracellular signal, regulated kinase 1/2 (ERK1/2), phospho-p38 MAPK, phospho-c-Jun N-terminal kinase (phospo-JNK) in jejunum/ileum and in Caco2 cells	Justino et al., 2015, 2020
**Prebiotics**		
Fructooligosaccharide (FOS)	Reduced MPO activity in jejunum section Decreased inflammatory infiltrate and preserved intestinal epithelium Attenuated weight loss and increased catalase levels	Smith et al., 2008; Galdino et al., 2018
**Synbiotics**		
Simbioflora^®^	Attenuated weight loss Improved histology of the intestinal mucosa and preserved epithelial architecture Reduced eosinophil infiltrate Decreased intestinal permeability Increased the production of extracellular factors, such as SCFA (acetate and butyrate)	Trindade et al., 2018
**Paraprobiotics**		
*L. rhamnosus* inactivated by heat	Prevented the expression of monocyte chemoattractant protein 1 (MPC-1) Regulated the expression of TNF-α, IL-12	Fang et al., 2014

In this context, studies conducted *in vitro* using Caco-2 cells (Fang et al., 2014) and *in vivo* with rats and mice have demonstrated strain-dependent effects of probiotics for the prevention/treatment of experimental mucositis induced by 5-FU, proving to be an effective therapeutic alternative for the treatment of this disease. Thus, they could be used in parallel with chemotherapy to promote the attenuation of gastrointestinal toxicity caused by cancer drugs, which is promising for improving the quality of life of patients undergoing chemotherapy treatment (Mi et al., 2017; Chang et al., 2018).

*Lactobacillus acidophilus* can decrease intestinal damage caused by 5-FU (applied at a dose of 450 mg/kg) by inhibiting the signaling of the NF-κB pathway, reducing levels of proinflammatory cytokines, such as TNF-α, IL-1β, and the C-X-C motif chemokine ligand 1 (CXCL-1); reversion in gastrointestinal dysmotility and increased gastric emptying and intestinal transit were observed (Justino et al., 2015). This probiotic was able to reduce inflammation and normalize bowel function in mice (Justino et al., 2015). Additionally, Oh et al. (2017) demonstrated that *L. acidophilus* A4 decreased the severity of intestinal mucositis induced by 5-FU (150 mg/kg) by stimulating overexpression of mucin genes (MUC2 and MUC5AC), reducing myeloperoxidase (MPO) activity, and inhibiting expression of proinflammatory cytokines, such as IL-1β, in mice (Oh et al., 2017).

*Lactobacillus casei* variety *rhamnosus* (*Lcr35*, Antibiophilus^®^, France) reduced the production of proinflammatory cytokines (TNF-α, IL-1β, IFN-γ, and IL-6), attenuated the loss of goblet cells, reduced the frequency of diarrhea, and restored the villus/crypt ratio, demonstrating an anti-inflammatory effect on tissue damage caused in the intestinal mucosa by administering 5-FU (30 mg/kg) for 5 days (Yeung et al., 2015). The protective effect of Lcr35 (1 × 10^7^ CFU) was also demonstrated in a colorectal cancer model; Balb/c mice were treated with a chemotherapeutic association called FOLFOX (30 mg/kg of 5-FU; 10 mg/kg of leucovorin, and 1 mg/kg of oxaliplatin) during 5 days (Chang et al., 2018). Lcr35 treatment was able to attenuate intestinal mucosa damage through regulation of the expression of proinflammatory cytokines (IL-1β, IL-6, TNF-α, and IL-10) induced by FOLFOX in the jejunum segment and also affected the gut microbiota composition, decreasing *Firmicutes* and increasing *Bacteroidetes* abundance (Chang et al., 2018). Thus, Lcr35 is a promising therapeutic strategy for the prevention or management of chemotherapy-induced intestinal mucositis (Chang et al., 2018).

A component in the intestinal microbiota, *Bifidobacterium bifidum* G9-1 (BBG9-1), has been widely used as a treatment for diarrhea and constipation, as well as for intestinal mucositis induced by 5-FU (50 mg/kg/6 days) (Kato et al., 2017). This probiotic can reduce diarrhea and interrupt weight loss, as well as being able to attenuate villus shortening and goblet cell degeneration. It can decrease inflammatory infiltrate in crypt cells, reduce MPO activity, reduce TNF-α and IL-1β levels, and also regulate the intestinal microbiota (decrease *Firmicutes* and increase *Bacteroidetes* abundance), demonstrating its ability to reduce the severity of 5-FU-induced intestinal mucositis (Kato et al., 2017).

Mi et al. (2017) demonstrated that *B. infantis* (1 × 10^9^ CFU/11 days) administration, in a synergic colorectal cancer treatment model with 5-FU (75 mg/kg/3 days) and oxaliplatin (8 mg/kg/3 days), was able to reduce the deleterious effects to the intestinal mucosa induced by chemotherapy. This probiotic improved the histology parameters, ameliorating the mucosal damage by decreasing Th1 and Th17 cells, and increasing the CD4^+^ CD25^+^ Foxp3^+^ Tregs response (Mi et al., 2017).

A combination of probiotic strains also demonstrated effectiveness in the reduction of intestinal damage induced by 5-FU chemotherapy. DM#1 mixture (*B. breve* DM8310, *L. acidophilus* DM8302, *L. casei* DM8121, and *Streptococcus thermophillus* DM8309) administration improved the restoration of the epithelial architecture, reduced neutrophil infiltration, reduced proinflammatory cytokines (TNF-α, IL-4, IL-6), and decreased intestinal permeability in mice treated with 5-FU (30 mg/kg/5 days) (Tang et al., 2017). Another study using a probiotic mix (*L. acidophilus*, *L. paracasei*, *L rhamnosus*, and *B. lactis*) showed that the mixture was able to prevent epithelial injury in intestinal mucositis induced by 5-FU (450 mg/kg), with an increase in the villus/crypt ratio and reduced malondialdehyde (MDA), MPO, TNF-α, and IL-6 levels in all small intestinal segments (duodenum, jejunum, and ileum) (Quaresma et al., 2019). In addition, administration of the probiotic mix resulted in an increase in glutathione (GSH) levels in the duodenum and jejunum sections and attenuated the delay in gastric emptying (Quaresma et al., 2019).

The therapeutic effects of probiotics also have been demonstrated for fermented products, which can be consumed by cancer patients. Milk fermented by *Lactobacillus delbrueckii* CIDCA 133 (7.5 × 10^7^ CFU) attenuated the damage caused to the intestinal mucosa by 5-FU (300 mg/kg), both in the recovery of the architecture of the epithelium, including prevention of goblet cell degeneration, and reduction of the polymorphonuclear cell infiltrate, with reduced IgA secretion and intestinal permeability (De Jesus et al., 2019).

A mulberry leaf extract fermented by *L. acidophilus* A4 strain stimulated overexpression of mucin genes (MUC2 and MUC5AC), promoted reduction of MPO, inhibited expression of proinflammatory cytokines, such as IL-1β, and reduced the loss of intestinal barrier function generated by 5-FU (150 mg/kg) administration (Oh et al., 2017).

The role of whey protein isolate (WPI) added to skim milk fermented by *Lactobacillus casei* BL23 (*L. casei* BL23) or by *Propionibacterium freudenreichii* CIRM-BIA138 (*P. freudenreichii* 138) was studied in a 5-FU-induced mucositis mouse model (Cordeiro et al., 2018). Milk fermented by both bacteria was sufficient to reduce weight loss, reduce histological scores, and prevent villus shortening and degeneration of goblet cells. WPI addition to fermented milk improved the effects of these probiotics, compared to when they were administrated alone (Cordeiro et al., 2018).

In addition to bacteria, yeasts can also have a beneficial effect on gastrointestinal mucositis. In this context, Porto et al. (2019) showed the effect of *Saccharomyces* cerevisiae UFMG A-905 alone or after enrichment with selenium, for intestinal mucositis treatment. This probiotic composition was able to preserve intestinal architecture and reduce nitrite concentration, lipid peroxidation, intestinal permeability, and inflammatory parameters, protecting mice against pathological consequences caused by 5-FU administration (Porto et al., 2019).

The probiotic, thermophilic, non-pathogenic yeast, *Saccharomyces boulardii*, was also tested for intestinal mucositis treatment; the histopathological changes caused by 5-FU were significantly reduced, including cell apoptosis and inflammatory parameters (nitrite concentration, neutrophil infiltrate, TNF-α and IL-1β cytokines, and CXCL-1 chemokine). This probiotic organism also improved the intestinal functions, such as gastric emptying, gastrointestinal transit, absorption, and intestinal permeability (Justino et al., 2015).

The effects of *S. boulardii* were evaluated by *in vitro* (Caco-2 cells treated with 1 mM 5-FU/24 h) and *in vivo* assays [Swiss mice treated with *S. boulardii* (1 × 10^9^ CFU/kg/3 days), mucositis induction by 5-FU (450 mg/kg)] (Justino et al., 2020). S. boulardii was able to modulate TLR2, TLR4, MyD88, NF-κB, ERK1/2, phospho-p38, phospho-JNK, TNF-α, IL-1β, and CXCL-1 expression, in these two different experimental models.

Based on the above studies, probiotics could be an effective therapeutic alternative for attenuating, preventing, and treating 5-FU-induced intestinal mucositis, although clinical studies will be required to test their safeness and usefulness for treatment.

## Prebiotics, Synbiotics, Paraprobiotics, and Postbiotics

The use of probiotics to treat intestinal mucositis is widely reported; however, research has also demonstrated the importance of fiber consumption to improve their benefit for the intestinal microbiota. These fibers are used by the microbiota organisms during the fermentation process, resulting in the production of various compounds, such as SCFAs, which are able to modulate the function of immune cells in the intestine, showing mainly anti-inflammatory effects (Tan et al., 2014; Luu and Visekruna, 2019).

To classify dietary fibers as prebiotic, it is necessary to satisfy six basic criteria: (i) they must be resistant to gastric acidity, hydrolysis by mammalian enzymes, and gastrointestinal absorption, (ii) they should not be digested in the upper gastrointestinal tract, (iii) they should be fermented in the colon by beneficial bacteria, (iv) they should be beneficial to the host’s health, (v) they should stimulate the growth of probiotics, and (vi) they should withstand food processing conditions while remaining unchanged (Wang, 2009; Markowiak and Ślizewska, 2017; Cerdó et al., 2019).

Prebiotics may be added to food or may be obtained through consumption of natural products, such as fruit, vegetables, cereals, and other edible plant products in which carbohydrate availability is high (Markowiak and Ślizewska, 2017). A wide variety of compounds have the potential to be classified as prebiotics. Most are non-digestible oligosaccharides extracted from plants, including fructooligosaccharide (FOS) (L’homme et al., 2003), galactooligosaccharide (GOS) (Ziegler et al., 2007), mannanoligosaccharide (MOS), and xylooligosaccharide (XOS) (Playne and Crittenden, 2002), oligofructose, and inulin (Roberfroid, 2007).

Prebiotic compounds stimulate growth, activating metabolism and promoting protection of bacteria that are beneficial to the host organism (e.g., saccharolytic bacteria, *Bifidobacterium*, and *Lactobacillus*). Prebiotic fermentation by indigenous microbiota can modulate the composition and the function of these microorganisms (Gibson and Roberfroid, 1995; Slavin, 2013; Davani-Davari et al., 2019). Furthermore, prebiotic fermentation can benefit the host through production of some compounds, such as SCFAs and lactic acid, produced by *Bifidobacterium* and *Lactobacillus* spp., which cause a reduction in the intestinal pH, inhibiting the development of gastrointestinal pathogens (Gibson and Wang, 1994; Bovee-Oudenhoven et al., 2003; Amani Denj et al., 2015). Prebiotics are also able to exert beneficial effects via mucin production by providing fermentable compounds that contribute to a lower incidence of bacterial translocation (Satchithanandam et al., 1990; Schley and Field, 2002).

Another mechanism proposed for prebiotics is their interaction with carbohydrate receptors (mannose, fucose and C-type lectin receptors, and galectins) on immune cells [phagocytes, natural killer (NK) cells, DCs]. The production of metabolites (e.g., folate and riboflavin, vitamins, and SCFAs) during their fermentation by gut microbiota showcases antimicrobial activity and maintains a healthy gut barrier (Hosono et al., 2003; Roller et al., 2004; Furusawa et al., 2013; Comstock et al., 2014; Levit et al., 2018; Enam and Mansell, 2019).

As prebiotics stimulate probiotic action, the synbiotic concept was created to overcome difficulties faced by probiotics in the GIT, demonstrating that this association (prebiotics + probiotics) intensifies their individual beneficial effects (Markowiak and Ślizewska, 2017).

Information on prebiotic stimulation of known probiotic strains leads to the choice of the ideal microorganism–substrate synbiotic pairs; the consumption of appropriately selected probiotics and prebiotics can increase the beneficial effects of each. Synbiotics have beneficial synergistic effects, greater than those observed for individual administration of prebiotics and probiotics (Geier et al., 2006).

The main criteria for synbiotic formulation should be a selection of appropriate probiotic and prebiotic pairs; the prebiotic should selectively stimulate the growth of probiotic microorganisms, having a beneficial effect on health, with no or limited stimulation of other microorganisms. The main probiotic species and prebiotics used in synbiotic formulations include, respectively, *Lactobacillus* spp., *Bifidobacteria s*pp., *S. boulardii*, and *B. coagulans*, and FOS, GOS, and XOS. The health benefits from the administration of synbiotics to humans include: (i) increased levels of *lactobacilli* and *bifidobacteria* and balanced gut microbiota, (ii) improvement of immunomodulating ability, (iii) prevention of bacterial translocation; and (iv) improvement of liver function and reduction of incidence of nosocomial infections in surgical patients (Pandey et al., 2015; Markowiak and Ślizewska, 2017). Evidence shows that physical and chemical changes in the colon and intestinal microbiota caused by synbiotic consumption, such as increased production of SCFAs and an increase in antitumor or antimutagenic compounds, can provide protection against rectal colon cancer, as they result in an improved immune response due to changes in the microbiota (Machado et al., 2014).

The studies listed above show the advantages of using live organisms; however, despite the fact that probiotics have proven benefits for the health of the host, current research emphasizes that the living organisms are not necessary for probiotic action; their different components, such as carbohydrates, proteins, lipids, vitamins, organic acids, cell wall components, and other complex molecules, generated after cell death, also have health benefits (Cuevas-González et al., 2020). The administration of non-viable organisms and their secreted products can present advantages in safety, reducing the possibility of infection and microbial translocation, which have been reported after the administration of probiotics to immunocompromised individuals (Aguilar-Toalá et al., 2018; Cuevas-González et al., 2020).

In this context, the terms “paraprobiotics” and “postbiotics” have been defined to refer to inactivated organisms and their metabolites. The difference between them is that paraprobiotics, also known as “non-viable probiotics” refer to inactivated cells, while postbiotics refer to soluble factors, which can be products (or metabolic byproducts) secreted by viable bacteria or released after their lysis (Cuevas-González et al., 2020). It is already possible to find products on the market that contain inactivated bacteria (e.g., Lactéol Fort^®^ from PUMC Pharmaceutical Co., Ltd. and Fermenti Lattici Tindalizzati^®^ from Frau, AF United Spa) (Taverniti and Guglielmetti, 2011).

Microorganisms can be inactivated through ultrasound (Ojha et al., 2016), high temperatures (Chuang et al., 2007), UV radiation (Lopez et al., 2008), and other options. However, it is necessary to evaluate some details to choose the best inactivation method, as well as to evaluate the effects on microbial structure and components (Ananta and Knorr, 2009; Taverniti and Guglielmetti, 2011).

The mechanism of action of paraprobiotics is not yet fully understood, but it is known that they are capable of acting in immunomodulation (Adams, 2010). *L.* rhamnosus GG (LGG), inactivated by UV radiation (Lopez et al., 2008) or heat killed (Li et al., 2009), has shown interesting results. UV-inactivated LGG is as effective as living LGG in downregulating the IL-8 response in Caco-2 cells; IL-8 is a proinflammatory chemokine released by intestinal cells (Lopez et al., 2008). Heat-killed LGG was tested in an infant rat model with LPS-induced inflammation and both live and inactivated strains administered enterally (10^8^CFU/kg); both were able to decrease proinflammatory mediators induced by LPS and to positively regulate anti-inflammatory mediators in the liver, plasma, and lung (Li et al., 2009).

The strains *L. acidophilus* A2, *L. gasseri* A5, and *L. salivarius* A6 inactivated by heat, in an *in vitro* experiment, were both able (at 10^5^ CFU/ml) to stimulate splenocyte and dendritic cell proliferation and production of IL-10, IL-12–p70, and IFN-γ. Likewise, *L. salivarius* was able to activate splenocytes and dendritic cells in mice to induce T cells toward a Th1 immune response. It was concluded that heat-inactivated bacteria can play an important role in modulating the immune response (Chuang et al., 2007).

A comparison was made of the *in vitro* potential of viable *L. rhamnosus*, the same bacteria inactivated by heat and the culture supernatant, for inducing the synthesis of cytokines by macrophages. Viable and heat-inactivated *L. rhamnosus* were able to induce the production of TNF-α, IL-6, and IL-10, demonstrating a capability to exert an immunoregulatory effect on macrophages (Jorjão et al., 2015).

Postbiotics is another term that emerged after it was found that not only live probiotic bacteria are capable of promoting health benefits. Postbiotics comprise all products obtained from the metabolic processes of live bacteria or released after bacterial lysis, with biological benefits for the host (Tsilingiri and Rescigno, 2013). These products include cell surface proteins (surface-layer proteins), cell-free supernatants (CFS), cell lysates, bacteriocins, enzymes such as glutathione peroxidase (GPx) and superoxide dismutase (SOD), peptides, teichoic acids, exopolysaccharides, B-group vitamins, secreted polysaccharides, organic acids (lactate), and SCFAs (acetate, propionate, and butyrate) (Tsilingiri and Rescigno, 2013).

Postbiotic mechanisms of action have not been fully elucidated; nonetheless, there is evidence that they promote antioxidant (Xu et al., 2011; Xing et al., 2015) and antiproliferative effects (Escamilla et al., 2012; Chuah et al., 2019), stimulating antipathogenic, immunomodulatory, and anti-inflammatory proprieties (Wang et al., 2018; Gao et al., 2019).

## Prebiotics, Synbiotics, and Paraprobiotics in Intestinal Mucositis

**FIGURE 5:**
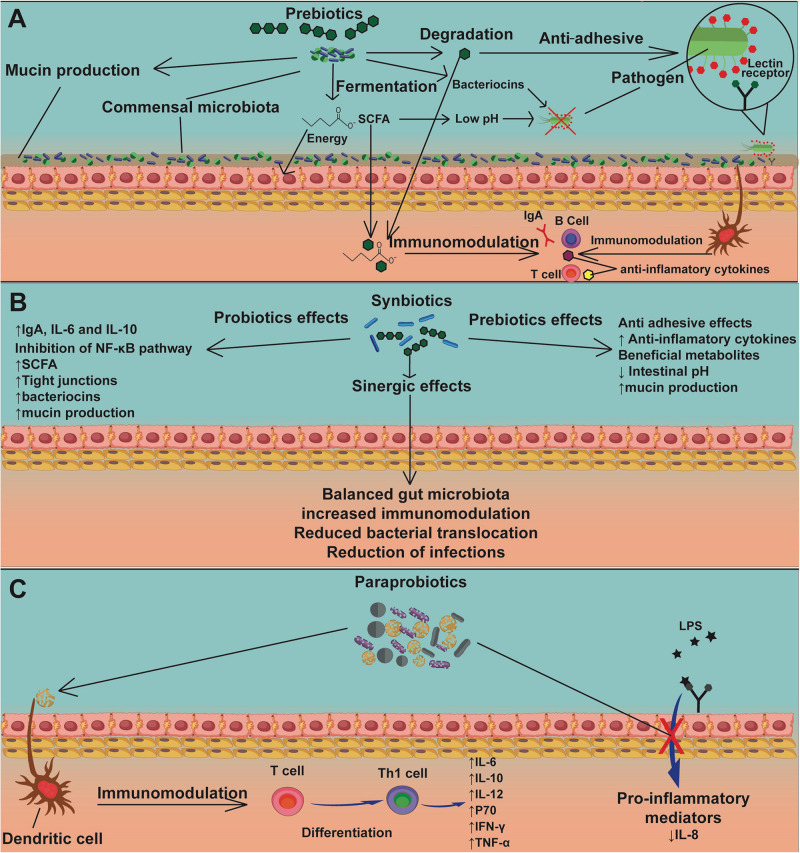
The mechanisms of action of prebiotics, synbiotics, and paraprobiotics. Prebiotics **(A)** act as nourishment for beneficial bacteria in the commensal microbiota, inducing the production of mucins, SCFAs, and bacteriocins, the latter two causing pathogen inhibition. Another mechanism by which prebiotics can inhibit pathogens is by interaction with an adhesion receptor, such as the lectin receptor, demonstrating an antiadhesive action. Sub-units of prebiotics and SCFAs can be used by the host cells for energy production and promote directly or indirectly, via dendritic cells, immunomodulation of lymphocytes, stimulating production of IgA and anti-inflammatory cytokines. Synbiotics **(B)** have mechanisms of action of both probiotics (Figure 4) and prebiotics **(A)**. Moreover, synbiotics have the advantage of generating a synergic effect, which promotes balance in the gut microbiota, increased immunomodulation, reduced bacterial translocation, and reduction of infections due to strong competition by probiotics against pathogens. The mechanism of action of paraprobiotics **(C)** is still not fully understood, though immunomodulation of T cells by dendritic cells has been reported, stimulating their differentiation into Th1 cells, promoting the production of anti-inflammatory cytokines. Another proposed mechanism is inhibition of signaling pathways related to LPS stimulation, resulting in a reduction of proinflammatory mediators, especially IL-8.

A few studies describe the action of prebiotics (Figure 5A), synbiotics (Figure 5B), and paraprobiotics (Figure 5C) on intestinal mucositis. Table 1 presents the main findings of their effects in intestinal mucositis. FOS supplement (3 and 6%) was administered to evaluate the effect on 5-FU (150 mg/kg)-induced intestinal mucositis in a murine model (Smith et al., 2008; Galdino et al., 2018). FOS was able to reduce MPO activity in a jejunum section. This was the only parameter that showed a significant reduction (Smith et al., 2008). In addition, beneficial effects of FOS (6%) administration in an experimental model of intestinal mucositis induced by 5-FU (300 mg/kg) were observed (Galdino et al., 2018). There was a decrease in inflammatory infiltrate, partial preservation of the intestinal epithelium, attenuation in body weight loss, and increased catalase levels, showing that supplementation with FOS could be an important adjuvant for the prevention and treatment of intestinal mucositis (Galdino et al., 2018).

Regarding the effects of synbiotics on intestinal mucositis, a commercial product called Simbioflora^®^, which is a synbiotic compound composed of 5.5 g of FOS plus four probiotic strains, *L. paracasei*, *L. rhamnosus*, *L. acidophilus*, and *B. lactis*, was evaluated (Trindade et al., 2018). This synbiotic was able to attenuate weight loss, decrease intestinal permeability, reduce eosinophil infiltrate, and also improve the histology of the intestinal mucosa, with preservation of the epithelial architecture, when compared to the administration of the isolated prebiotics (Trindade et al., 2018). In addition, it was found that this synbiotic increases the production of extracellular factors, such as SCFAs (acetate and butyrate), which could contribute to the observed immunomodulating activity (Trindade et al., 2018).

The effects of paraprobiotics on mucositis were demonstrated by Fang et al. (2014). To examine the immunomodulatory properties of *L. rhamnosus*, the bacteria were inactivated by heat and evaluated in an *in vitro* model of intestinal mucositis using Caco-2 cells (Fang et al., 2014). This revealed that heat does not affect the cell integrity of this bacterial species, maintaining its rod-shaped structure intact, considerably reducing the expression of monocyte chemoattractant protein 1 (MCP-1), and regulating the expression of TNF-α and IL-12. The same results were obtained with live bacteria, revealing that this bacterial species conserved intact probiotic properties after heat inactivation, making it a promising candidate for further studies (Fang et al., 2014).

In a study of the postbiotic effect on 5-FU-induced intestinal mucositis, Prisciandaro et al. (2011) found that *Escherichia coli Nissle* 1917 (EcN) supernatant partially protected the mouse intestine from 5-FU damage (150 mg/kg) (Prisciandaro et al., 2011). It was observed that this postbiotic was able to help avoid histological damage (villus height and crypt depth) and prevented a decrease in acidic mucin-producing goblet cells. Another study showed that oral butyrate supplementation (9 mM) was able to reduce the damage to the intestinal mucosa caused by this antineoplastic agent (200 mg/kg). Reduction in histological damage, ulceration, and amelioration in intestinal permeability were observed. The gene expression of the *tight junction* protein ZO-1 (zonulin) was increased, and proinflammatory cytokines, such as TNF-α and IL-6, were reduced (Ferreira et al., 2012).

The supernatant of mulberry leaf extract fermented by *L. acidophilus A4* was able to reduce gene expression of proinflammatory cytokines IL-1β and myeloperoxidase (MPO), and stimulate overexpression of mucin genes (MUC2 and MUC5AC), thus reducing the severity of intestinal mucositis induced by 5-FU (150 mg/kg) (Oh et al., 2017). Additionally, *Lactobacillus plantarum* supernatant inhibited the expression of the specific markers CD44, CD133, CD166, and ALDH1 of 5-FU-resistant colorectal cancer cells (CRC) (HT-29 and HCT116) (An and Ha, 2016). The combination therapy of this postbiotic and 5-FU induced an anticancer mechanism by inactivating the Wnt/β-catenin signaling of chemoresistant CRC cells and led to cell death by inducing caspase-3 activity. These results suggest that probiotic secretory substances can regulate cell proliferation in colorectal cancer and may be a therapeutic alternative for treating chemoresistant colorectal cancer (An and Ha, 2016).

To date, there have been few rigorous investigations examining the effect of prebiotics on 5-FU-induced intestinal mucositis. Knowing its potential in the intestinal mucosa, their supplementation with probiotics may be an attractive therapeutic alternative to ameliorate symptoms caused by mucositis, as well as other diseases involving the GIT.

Despite the significant impact of mucositis and advances in research to understand this pathology, existing therapies are mainly limited to clinical management of symptoms, aiming at electrolyte replacement, oral rehydration, and the use of adjuvant agents, such as loperamide octreotide, sucralfate enemas, sulfasalazine, and hyperbaric oxygen, to reduce fluid loss and decrease intestinal motility and diarrhea associated with mucositis, which are important debilitating symptoms (Van Sebille et al., 2015; Ribeiro et al., 2016). Given that it is necessary to find more effective therapeutic alternatives to combat intestinal mucositis, the “biotics” are strong candidates.

## Final Considerations

The antineoplastic drug 5-FU is an essential and useful option for cancer treatment; however, its side effects, especially mucositis, can complicate treatment continuity and may lead to death. Effective measures to combat these symptoms, improving the quality of life of cancer patients, are crucially needed.

The probiotics have been investigated in various studies because of their beneficial properties for the GIT, including attenuation of dysbiosis. Several probiotic bacteria studied in intestinal mucositis murine models were able to attenuate and prevent intestinal histological damage, and also decrease weight loss and proinflammatory cytokine secretions, proving to be quite efficient in ameliorating the side effects to the intestine caused by 5-FU.

Though they can improve the health of the host, administration of viable microorganisms to immunosuppressed individuals still leads to controversial clinical findings. Paraprobiotics could be an effective alternative to address this concern, since microbial cells are dead or inactivated, thus avoiding risks associated with their administration to immunocompromised individuals.

Prebiotics are also described in the literature for their regulatory ability, acting to modify the commensal microbiota to a beneficial state. However, there are a few studies evaluating their potential for helping avoid intestinal mucositis. The existing studies demonstrate that prebiotics, when associated with a probiotic, are more efficient than when they are used separately, attenuating the symptoms of mucositis and improving to almost normal status the histology of the GIT.

Therefore, probiotics, prebiotics, synbiotics, paraprobiotics, and postbiotics may be useful alternatives for the treatment of intestinal mucositis induced by 5-FU. However, further studies are needed to elucidate all of the mechanisms of action of these bacteria and prebiotics to evolve into human clinical trials.

## Author Contributions

VB, TS, LT, LJ, FB, and NC-R wrote the original draft of the manuscript. VA, MD, and PM-A reviewed and revised the manuscript, obtained funding, and supervised the project. All authors contributed to the article and approved the submitted version.

## Conflict of Interest

The authors declare that the research was conducted in the absence of any commercial or financial relationships that could be construed as a potential conflict of interest.
